# Supplementary material for: SPEED (SPlEnic Embolisation Decisions) study—Decision to treat acute traumatic splenic artery injury in the context of trauma protocol
Source: PLoS One. 2025 Jan 8;20(1):e0313138. doi: 10.1371/journal.pone.0313138 (PMC11709302; doi:10.1371/journal.pone.0313138)
Supplement: S1 Appendix — (DOCX) [file pone.0313138.s001.docx]

**List of UK Major Trauma Centres (MTCs)**

**Adult and Children’s Major Trauma Centres**

1. Addenbrooke’s Hospital Cambridge
2. Frenchay Hospital Bristol
3. James Cook University Hospital, Middlesborough
4. John Radcliffe Hospital Oxford
5. King’s College Hospital London
6. Leeds General Infirmary
7. Queen’s Medical Centre Nottingham
8. Royal London Hospital
9. Royal Victoria Infirmary Newcastle
10. St Mary’s Hospital London
11. St George’s Hospital London
12. Southampton General Hospital

**Adult Major Trauma Centres**

1. Derriford Hospital Plymouth
2. Hull Royal Infirmary
3. Northern General Hospital Sheffield
4. Queen Elizabeth Hospital Birmingham
5. Royal Preston Hospital
6. Royal Sussex County Hospital Brighton
7. University Hospital Coventry
8. University Hospital of North Staffordshire Stoke on Trent

**Collaborative MTCs**

22. Manchester Collaborative MTC

a) Salford Royal NHS Trust

b) Manchester Royal Infirmary

c) University Hospital South Manchester

23. Liverpool Collaborative MTC

a) Aintree University Hospital

b) Walton Centre

c) Royal Liverpool University Hospital
